# Search for Effective Approaches to Fight Microorganisms Causing High Losses in Agriculture: Application of *P. lilacinum* Metabolites and Mycosynthesised Silver Nanoparticles

**DOI:** 10.3390/biom12020174

**Published:** 2022-01-21

**Authors:** Masudulla Khan, Azhar U. Khan, Mohd Rafatullah, Mahboob Alam, Nina Bogdanchikova, Diana Garibo

**Affiliations:** 1 Botany Section, Women’s College, Aligarh Muslim University, Aligarh 202002, India; masudkhann@gmail.com; 2 School of life and Basic Sciences, Department of Chemistry, SIILAS CAMPUS, Jaipur National University, Jaipur 302017, India; azhar.u.kh@gmail.com; 3 School of Industrial Technology, Universiti Sains Malaysia, Gelugor 11800, Penang, Malaysia; mrafatullah@usm.my; 4 Division of Chemistry and Biotechnology, Dongguk University, 123, Dongdaero, Gyeongju-si 780714, Korea; mahboobchem@gmail.com; 5 Centro de Nanociencias y Nanotecnología, Universidad Nacional Autónoma de México, Ensenada 22800, Mexico; 6 Centro de Nanociencias y Nanotecnología, CONACYT Research Fellow at Universidad Nacional Autónoma de México, Ensenada 22800, Mexico

**Keywords:** *Purpureocillium lilacinum*, *Aspergillus flavus*, *Meloidogyne incognita*, AgNPs

## Abstract

The manuscript presents the first report to produce silver nanoparticles (AgNPs) using soil-inhabiting *Purpureocillium lilacinum* fungus cell filtrate as a promising fungicide and nematicide on two microorganisms causing high economic losses in agriculture. Methods: A fungus biomass was used as a reducing and stabilising agent in the process of NPs synthesis and then characterisation done by SEM, TEM, UV-Vis. Finally, the antimicrobial activity of the synthesised AgNPs was determined. Results: Synthesised AgNPs with a spherical and quasi-spherical shape with an average diameter of 50 nm were effective to inhibit *A. flavus* fungi and *M. incognita* root knot nematode, which are extremely pathogenic for plants. Application of the AgNPs led to 85% reduction of proliferation of *A. flavus*, to a 4-fold decrease of hatching of *M. incognita* plant-parasite juveniles from eggs, and to a 9-fold increase of *M. incognita* nematode mortality. Conclusions: Biosynthesised AgNPs can be used as an effective fungicide and nematicide for food safety and security and improvement of agricultural production, but further agricultural field trials are required to observe their effect on environment and other factors.

## 1. Introduction

Many microorganisms cause diseases in crop plants, affecting crop production and leading to huge yield losses. Plant parasitic nematodes cause high losses in agriculture, from around $US80 billion to $US157 billion in crop losses worldwide [1]. Root knot nematode *Meloidogyne* *incognita* is a plant parasitic nematode that causes root knot disease in many important crop plants such as tomatoes, lettuces, peppers, etc. [1,2]. Additionally, the plant fungal diseases represent a huge problem in food safety concerns. The filamentous fungus *Aspergillus flavus* is a pathogenic and saprophytic fungus that produces aflatoxins causing disease in crops such as maize, cottonseed, and peanuts, reducing crop yield [3]. In addition, this fungus causes intoxication in humans by the consumption of nuts, grains, and their derived products contaminated by the fungus [4]. Due to *A. flavus* infection to the important crops and aflatoxin in grains, this contamination causes high losses in agriculture, so that hundreds of million dollars are lost to the U.S. and world economy annually. To manage yield losses and to improve global food security it is necessary to improve agricultural production. Conventional methods such as chemical pesticides are not enough to control the pathogenic microbes so there is a continuous search for novel and highly effective approaches for their control. Nanomaterials provide new perspectives, but at present they are in the nascent stage and need more research to understand their impact on environment and human health. Nanomaterials, especially nanoparticles (NPs), attract attention for agriculture problems due to their effective bactericidal, fungicidal, and nematicidal activity [5].

The different metals including silver, copper, titanium, zinc, and gold are used to produce NPs with antimicrobial properties [6]. Biologically synthesised silver nanoparticles (AgNPs) have many applications [7]; AgNPs are widely used in various fields for bio-labeling, as antimicrobial agents, sensors and filters, in microelectronics, and catalysis [8]. Meanwhile, biological methods for nanoparticles synthesis are considered eco-friendly, safe, cheap, clean, and non-toxic, thus are recommended as good alternatives to chemical and physical methods. The most-often used biological entities are plants, algae, bacteria, fungus, actinomycetes, yeast, and viruses [9,10,11]. In addition, usually in research the effectivity of nanoparticles synthesised using plant extracts or microorganism metabolites is investigated, while the properties of the extracts and solutions of metabolites themselves are not investigated. Thus, very little attention is paid to the activity of these solutions in the fight against those microorganisms causing high losses in the agriculture.

Fungi produce many compounds that could have various applications. Approximately 6400 bioactive compounds are produced by fungi so they could be used as reducing and stabilising agents. Furthermore, fungi could be cultivated easily on a large scale and can produce nanoparticles with controlled shape, size and morphology. Fungi have advantages over other microbes, in that they produce large quantities of enzymes, proteins, and other compounds. Some compounds could play important role in sustainable synthesis of NPs [12]. The first use of fungi to synthesise NPs dates back to a letter in *Nature* in 1989, where *Candida albicans* was used to produce Cd-Se NPs [13]. Since then, many fungi that synthesise metal NPs have been reported to mycosynthesise metal NPs [14].

In previous studies, we have synthesised AgNPs using plant biomass [11,15], but in the present study, we have evaluated the activity of the non-toxic species of fungus *Purpureocillium lilacinum* in the biosynthesis of AgNPs and their toxic effect on the fungus *A. flavus* and the root knot nematode *M. incognita*. In the past, *P. lilacinum* was known as a fungus that can cause disease in humans [16]. However, in recent decades it has been an effective nematophagous, not pathogenic for humans. So, it could be used as a biocontrol agent against the plant parasite *M*. *incognita* [17] by using the cell filtrate of this fungus for the biosynthesis of AgNPs. Due to the easy processes of *P. lilacinum* isolation and mass production, it also plays an important role in the management of horticultural pests [18].

In order to fully utilise the advantage of nanotechnology in plant disease protection and management, we report a green, handy and environment friendly approach for the biosynthesis of AgNPs using *P. lilacinum* as a bioresource, which is non-hazardous and inexpensive. Then, biosynthesised AgNPs were characterised. Additionally, we evaluated their toxic effect on the fungus *A. flavus* and the root knot nematode *M. incognita* microorganisms which cause significant economic implications for the agricultural industry worldwide. Or, in other words, to put the non-pathogenic fungi strain into service against pathogenic species.

## 2. Experimental Design

### 2.1. Culture Conditions for Microorganism

*P*. *lilacinus* was cultured in Potato Dextrose Agar (PDA) media and incubated at 26–28 °C for further use. Fungal mycelium and conidia were studied for species’ confirmation under a light microscope, and fungus sub-culturing was used for the experiment. *A*. *flavus* was also cultured on PDA medium for further use, and the root knot nematode *M*. *incognita* was isolated from infected roots of brinjal, as we explained in previous studies [2,15].

### 2.2. Biosynthesis of AgNPs

**Figure 1:**
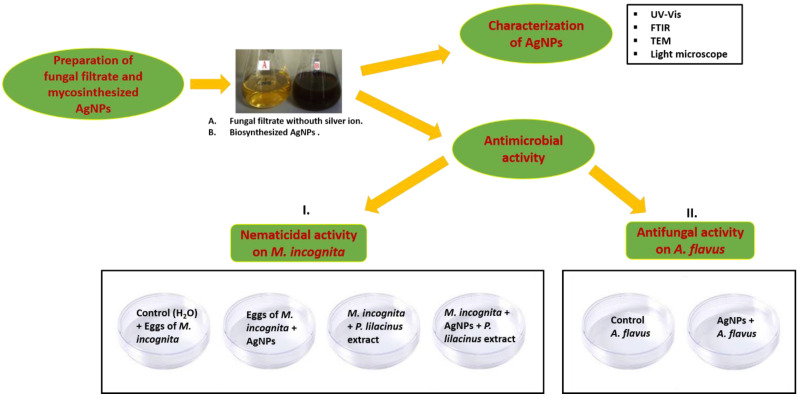
Graphical scheme of study design.

Experiments were conducted with the *P*. *lilacinus* fungal cell filtrate used to prepare AgNPs (Figure 1). To obtain enough amount of fungal biomass, the fungus was cultivated in broth liquid media. Flasks with fungus culture were incubated at 24 ± 2 °C for 7–8 days. After the incubation, 2 g of fungal biomass was isolated and mixed with 100 mL of Milli-Q deionised water for 48 h at 28 °C and 150 rpm. Silver nitrate (AgNO_3_, GR) was purchased from Merck, India to prepare a 1 mM solution of AgNO_3_ in an Erlenmeyer flask. After filtration of the fungal extract using a Whatman filter paper No.1, 50 mL of a 1 mM AgNO_3_ solution was mixed with 50 mL of cell filtrate in a 250 mL Erlenmeyer flask, and was kept on a shaker at 150 rpm for 48 h. The synthesised AgNPs had a concentration of 54 ppm of metallic silver content. Fungal biomass filtrate without adding the AgNO_3_ solution was used as control. The reduction of silver metal ions was monitored by visual inspection of the solution’s colour change from yellowish to dark brown. The formation of AgNPs was measured in an ultraviolet-visible spectrophotometer in the range of 150–550 nm. Prepared AgNPs were subjected to different techniques for further characterisation.

### 2.3. Characterisation of Biosynthesised AgNPs

The formation of the reduced AgNPs in solution was monitored by using a Shimadzu UV–Vis spectrophotometer (UV-1800, Japan). The absorption spectra were registered between 200 and 800 nm and deionised water as the blank. The Fourier Transform Infrared Spectroscopy (FTIR) spectrum of the biosynthesised silver product was recorded on a FTIR spectrometer between 400 and 4000 cm^−1^ at a resolution of 4 cm^−1^. Transmission Electron Microscopy (TEM) was performed after drying a drop of aqueous AgNPs on carbon-coated copper grid. Afterward samples were dried and kept under vacuum in desiccators before loading on a TEM specimen holder. TEM analysis was done in the same way as explained in our previous study [15]. The particle morphology and size distribution of biosynthesised NPs were evaluated.

### 2.4. Toxic Effect of Biosynthesised AgNPs on Nematode

*P*. *lilacinum* metabolite-containing AgNPs were used for screening their nematicidal activity against *M*. *incognita* plant pathogenic nematode (I in Figure 1). To determine the nematicidal activity of the prepared AgNPs, a 5 mL (54 ppm nominal concentration of metallic silver) suspension was dissolved in 15 mL of double distilled water in Petri dishes. Ten nematode eggs were placed in each Petri dish with 50 mg of *P*. *lilacinus* fungus in 20 mL of double distilled water. Similarly, another Petri dish had ten eggs, 50 mg of *P*. *lilacinus* fungus and 5 mL of 5.39 × 10^−^^3^ cells prepared with AgNPs in 15 mL of double distilled. A Petri dish with ten eggs and 20 mL of double distilled water was used as a control (without *P*. *lilacinus* and AgNPs). Then, 5.39 × 10^−3^ cells solutions were prepared by dissolving 0.1 mL of a solution of synthesised AgNPs in one litre of distilled water. The effect on nematode hatching was observed for 24 and 48 h and mortality for 48 h. All the tests were run in triplicate and the average results were taken.

### 2.5. Toxic Effect of AgNPs on the Fungus

Biosynthesised AgNPs stabilised with the fungal cell filtrate were used to screen for in vitro antifungal activities against the plant pathogenic fungus *A*. *flavus* (II in Figure 1). Fungus incubated in PDA medium at 25 ± 2 °C for 10–12 days with 54 ppm of AgNPs was used to screen the effect of AgNPs on mycelia growth, compared with the control (without AgNPs) and three experiments in triplicate were done. Antifungal activity was measured, as described in our previous studies [15,19].

### 2.6. Statistical Analysis

Statistical analysis of results to analyse the significance was done by using MS Excel and R software (Agricolae, 3.6.1).

## 3. Results

### 3.1. P. lilacinum Fungus Culture, Conidia, and Mycelium

**Figure 2:**
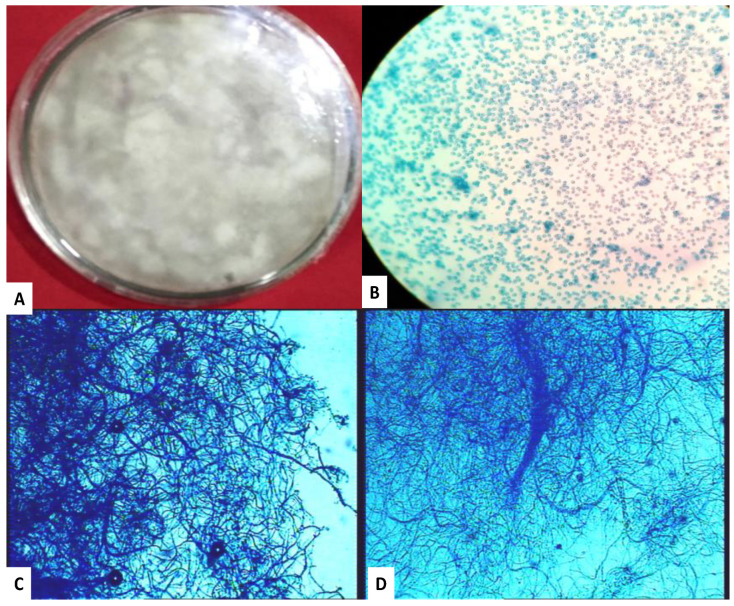
Light microscopic images of (**A**). *P. lilacinus* fungus culture on PDA media; (**B**). Conidia of fungus; (**C**,**D**). Mycelium of fungus.

*P. lilacinum* conidia and mycelium were studied under a light microscope (Figure 2). The photograph in Figure 2A shows the aerial mycelium of *P*. *lilacinum* fungus cultured on solid PDA medium. Conidia and pure mycelium were obtained by growing the fungus on a solid medium under static growth conditions (Figure 2B–D).

### 3.2. UV-Visible Spectroscopy

**Figure 3:**
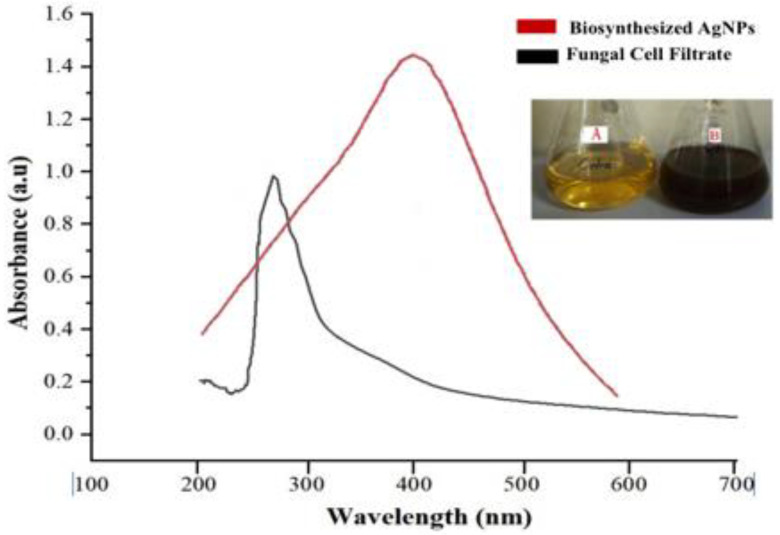
UV–vis absorption spectra of fungus cell filtrate and biosynthesised AgNPs. (**A**). Aqueous extract of *P*. *lilacinum;* (**B**). Reaction mixture containing AgNO3 and *P*. *lilacinum* extract.

AgNPs formation was first identified by a visual colour change in the reaction mixture containing AgNO_3_ and fungal biomass over 1 h (Figure 3A,B). The colour change in the suspension helped to determine the presence of silver-reduced species. Figure 3 shows the UV-Vis spectra (wavelengths ranging from 200 to 800 nm with a resolution of 1 nm) recorded for synthesised AgNPs. The maximum peak position in the spectrum of the biosynthesised Ag species was observed at 410 nm (Figure 3), which is typical peak for surface plasmon resonance of AgNPs.

### 3.3. Fourier Transform Infrared Spectroscopy

**Figure 4:**
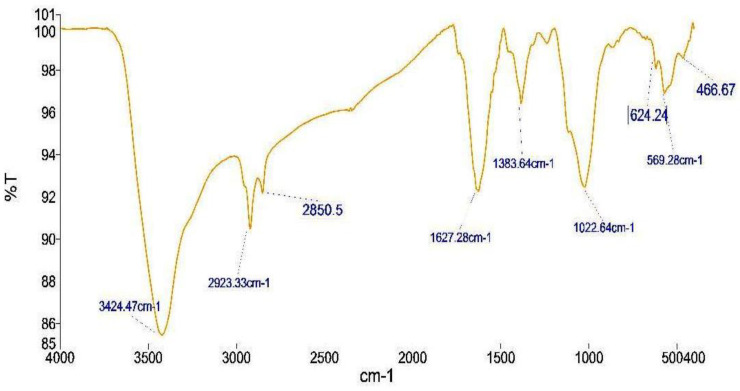
FTIR of biosynthesised AgNPs.

The FTIR spectroscopy analysis was performed to identify functional groups of the fungal filtrate responsible for the reduction of Ag^+^ ions, serving as capping/stabilisation agents of bio-reduced AgNPs (Figure 4).

In the FTIR spectrum, peaks at 3424, 2923, 1627 and 1383 cm^−1^, were attributed to: (1) vibrations of OH-groups, (2) CH and CH_2_ stretching vibrations, (3) C=O stretching, and (4) aromatic ether C–O–C, phenolic C–O, and ester C–O–O–C stretching vibrations, respectively (Table 1). The broad band interval at 3350–3450 cm^−1^ indicates the –OH stretching vibrations of the hydroxyl groups corresponding to H-bonded alcohols and also to intramolecular H bonds, which are most probably from water molecules. The peaks at 1620–1670 prove the existence of enzymes and/or proteins. These peaks at 1350–1520 suggested the presence of aromatic and/or phenolic compounds in the fungal extract. The FTIR of biosynthesised AgNPs indicates the dual role of the *P. lilacinum* fungus filtrate as a reducing agent and as a stabilising agent.

### 3.4. TEM Analysis

**Figure 5:**
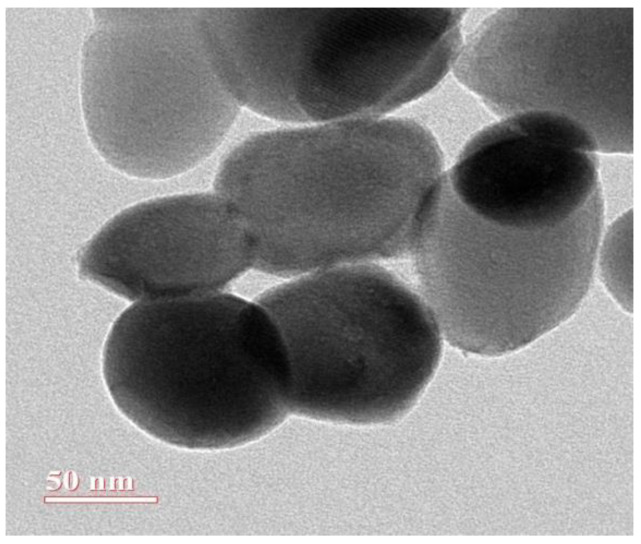
TEM image of synthesised AgNPs.

TEM was carried out to investigate the morphology of biosynthesised AgNPs. Figure 5 shows the presence of spherical and quasi-spherical particles with an average diameter of 50 nm (Figure 5).

### 3.5. Effect of AgNPs on A. flavus Fungi

**Figure 6:**
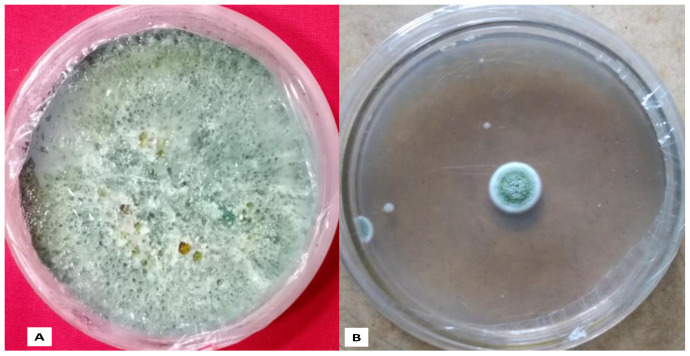
(**A**). Untreated culture of *A. flavus;* (**B**). *A. flavus* culture treated with AgNPs.

**Figure 7:**
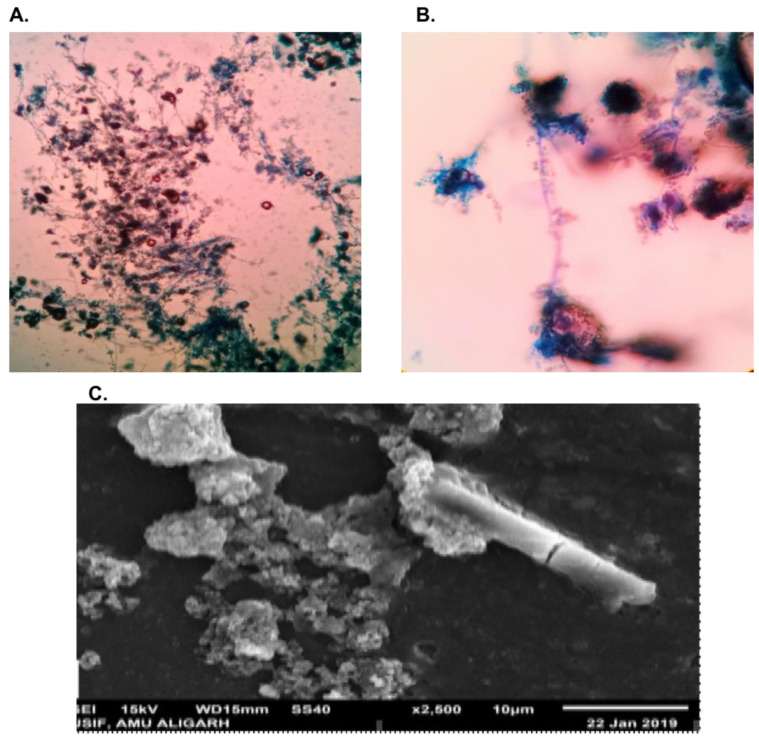
(**A**,**B**). Light microscope images of *A*. *flavus* fungal mycelium treated with AgNPs; (**C**). SEM image showing disturbed fungal mycelium treated with biosynthesised AgNPs.

The antifungal effect of AgNPs was investigated on *A. flavus* using the agar diffusion of their mycelium. Figure 6 shows in vitro results of the effect of AgNPs on mycelia growth (Figure 6B compared to the control (without AgNPs) (Figure 6A). Growth of *A*. *flavus* was considerably reduced to 85% in the PDA medium with 54 ppm AgNPs compared with the control (without NPs) (Figure 6 and Figure 7). Microscopic and SEM studies showed that the fungal mycelium were disturbed by the synthesised AgNPs (Figure 7). Inhibition of fungal growth calculation was explained in our previous study [15].

### 3.6. Effect of AgNPs on M. incognita Nematodes

**Figure 8:**
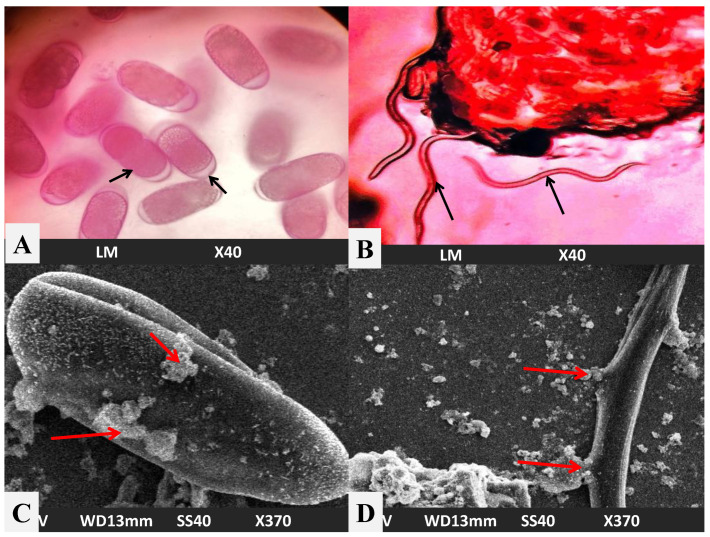
Light microscope images: (**A**). untreated eggs of *M. incognita*; (**B**). untreated second-stage juveniles of *M. incognita* nematode. SEM images of nematode *M. incognita* treated with AgNPs; (**C**). Eggs; (**D**). second-stage juvenile *M. incognita* nematode. Red arrows indicate zone of contacts of the AgNPs with the *M. incognita* eggs and second-stage juvenile nematode.

Untreated *M. incognita* nematodes and eggs are shown in Figure 8A,B. SEM images show that the cells treated with AgNPs suffered some changes and possible damage on the cell surface, because they are in contact with the *M. incognita* eggs and second-stage juvenile nematodes (Figure 8C,D).

**Figure 9:**
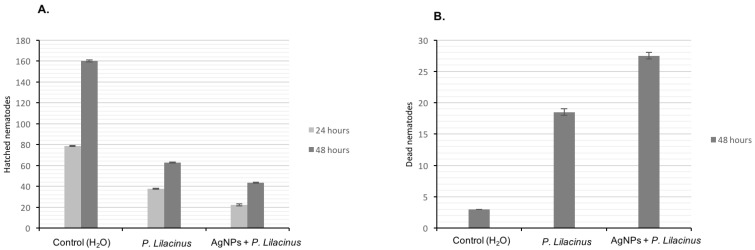
In vitro effect of *P. lilacinum* filtrate and biosynthesised AgNPs stabilised with this filtrate: (**A**) number of eggs hatching after 24 and 48 h, (**B**) number of dead *M. incognita* nematodes after 48 h.

In vitro studies showed that the *P. lilacinum* filtrate itself and the synthesised AgNPs have nematicidal activity and inhibit the hatching of *M*. *incognita* from eggs (Figure 9).

After 24 h hatching of nematodes in double-distilled water (control) were approximately 2 and 4 times higher than after the treatment with *P. lilacinum* filtrate and with AgNPs, respectively (Figure 9A). It implies that the *P. lilacinum* filtrate itself has nematicidal activity against *M*. *incognita* eggs, and that the nematicidal activity increased twice when this filtrate contained AgNPs. The number of hatched nematodes after 48 h increased 1.5–2 times compared to 24 h (Figure 9A). After 48 h, the lowest death count of nematodes was in double-distilled water (control) (Figure 9B). It increased 6 and 9 times after the treatment with the *P*. *lilacinum* filtrate and AgNPs, respectively. Hence, despite the fact that the concentration of the AgNPs was extremely low (0.0054 wt.%), but their addition to *P*. *lilacinum* filtrate led to significant increase of activity against *M*. *incognita* eggs and nematodes.

The results of the present study showed that the mycosynthesis of AgNPs have nematicidal and antifungal activity in vitro against *M. incognita* and *A. flavus*, respectively. This finding is consistent with previous studies which showed that AgNPs are effective for killing fungi (Table 2) [24,25,26].

## 4. Discussion

The present work presents a method of an extracellular mycosynthesis of AgNPs using the metabolites of the fungus *P*. *lilacinum*, which, as far as we know, is the first report dedicated to eco-friendly synthesis of nanoparticles using *P. lilacinum* fungus cell filtrate. Extracellular metabolites play an important role because phenols can reduce the precursor salt (AgNO_3_) and microorganism proteins work as AgNPs’ stabilisers [12]. Methanol extracts of the secondary metabolites of *P*. *lilacinum* show the presence of other components such as fatty acids (approx. 82%) that participate as insecticidal and nematicidal agents [26]. Our obtained results clearly demonstrated that the *P*. *lilacinum* metabolites have a nematicidal effect 1.4 times less than the biogenic AgNPs.

There are several works that use the secondary metabolites of non-pathogenic fungi to produce AgNPs. Banu et al., 2014 synthesised AgNPs from the fungus *Beauveria bassiana,* which were effective against the dengue vector *Aedes aegypti* (the yellow fever mosquito) [26]. Bhainsa et al., 2006 synthesised AgNPs from *Aspergillus fumigates* fungus [28]. Li et al., 2012 synthesised AgNPs from fungus *Aspergillus terreus* [27]. Raheman et al., 2011 reported the synthesis of AgNPs using endophytic fungi *Pestalotia* sp., isolated from *Syzygium cumini* leaves and tested for antibacterial properties against *Staphylococcus aureus* and *Salmonella typhi* [29]. Gade et al., 2008 reported the biosynthesis of AgNPs using the fungus *Aspergillus niger* [30]. However, none of them have used *P*. *lilacinum* for the synthesis of AgNPs, despite being used as a biocontrol in plants.

The use of AgNPs as antimicrobial agents has detrimental effects on fungal hyphae and conidial germination of fungus [31]. Mishra et al., 2014 synthesised AgNPs by using the supernatant of a culture of an agriculturally essential bacterium, *Serratia* sp. BHU-S4 and showed their important role for the management of spot blotch disease in wheat. The synthesised AgNPs showed efficient antifungal potential against the fungus *Bipolaris sorokiniana,* which causes this disease [32]. Gupta and Chauhan [33] highlighted the fungicidal property of AgNPs against fungus *Alternaria brassicicola* that causes Black Spot in cauliflower, cabbage, radish, and kale, which results in severe yield loss. They tested AgNPs at 10, 25, 50 and 100 ppm concentrations against *A. brassicicola* cultured on PDA in Petri dishes. They calculated the inhibition percentage in fungal growth with a view to investigate the antifungal ability of AgNPs. Treatment with 100 ppm AgNPs produced the maximum growth inhibition of the fungus *A*. *brassicicola* [33]. To our knowledge, no works that use *P*. *lilacinum* to produce AgNPs with a toxic effect on *M. incognita* have been reported.

Regarding *A. flavus*, there are few studies where the effect of AgNPs on its growth was evaluated. Not all of them have mentioned the AgNPs concentrations necessary for complete inhibiting effect on *A. flavus* [24,25,27], which makes it difficult to compare the inhibition potential of biosynthesised AgNPs described in these publications. We have found only one publication, where Bocate et al., 2019 reported a MIC of less of 50% of the mycelium at 8 ppm of AgNPs [24]. In our work, however, AgNPs were able to inhibit approximately 85% of the mycelium at the concentration of 54 ppm. These results can be compared as they are presented now. For their comparison it would be necessary to measure the curve of inhibiting capacity of our AgNPs with their concentration in future work. More recently a work with chemically synthesised AgNPs showed that at a higher concentration of AgNPs (100 ppm) only 39% of the mycelium was reduced without eliminating the fungus [34]. This leads to the hypothesis that synthesis with application of the secondary metabolites of *P. lilacinum* fungus and Ag+ ions permitted to obtain biogenic AgNPs with increased potential for antifungal application. The concentrations of AgNPs used and their promising antifungal effects indicate on the importance of study of these AgNPs toxicity for humans, which needs to be fully studied in a future work.

The physicochemical characteristics of AgNPs influence their antimicrobial effect. Ibrahim (2015) found that AgNPs have high antimicrobial properties compared to other NPs which may be due to their extremely large surface area, providing an efficient attachment with the microorganism cell wall [35]. Vahabi et al., 2011 observed that the antifungal activity of AgNPs may be due to the suppression of enzymes and toxins used by fungi for pathogenesis [36]. Although there are various works that suggest another mechanism of AgNPs activity: DNA disruption, membrane disruption, reactive oxygen species (ROS), the mechanism is not fully elucidated for the specific microorganism studied here. Nevertheless, a deep evaluation is required to understand detailed mechanisms of antifungal action of AgNPs. The results obtained in the present article showed that the biogenic AgNPs have a potential for being used in the improvement of agricultural production.

## 5. Future Prospective and Safety Measure

Systematic studies on identification of compounds in the *P. lilacinum* extract and detailed physicochemical characterisation, storage stability and influence of pH, temperature, etc. for biosynthesised AgNPs should be performed in future. It is important because this work is just a first approach showing a potential for synthesised AgNPs to be used against agricultural pathogens. Usually in research the effectivity of nanoparticles synthesised using plant extracts or microorganism metabolites is investigated, while the properties of the extracts and solutions of metabolites themselves should be investigated too. The studies of toxicity of biosynthesised AgNPs are also needed to understand their effect on environmental, crop production (fruits, vegetables, etc. for human consumption), and humans before their mass production and application in agriculture.

## 6. Conclusions

Applying the soil-inhabiting *P. lilacinum* fungus cell filtrate as a reducing and stabilising agent, the synthesis of AgNPs was achieved. The biosynthesised AgNPs showed antifungal activities against two microorganisms (A. *flavus* fungi and *M. incognita* root knot nematode), which are extremely pathogenic for plants. The application of the AgNPs reduced 85% of *A. flavus* proliferation, led to a 4-fold decrease of hatching of plant-parasitic *M. incognita* juveniles from eggs and to a 9-fold increase of *M. incognita* nematode mortality. It was shown that *P. lilacinum* fungus cell filtrate, along with their functions as a reducing and stabilising agent, possesses also antifungal activities against *M. incognita*, which increased when the filtrate is combined with AgNPs. In the present work, the AgNPs activity against *A. flavus* was at least 1.7 times higher than the activity of AgNPs studied in previous research. The obtained results demonstrated that biosynthesised AgNPs have potential to be used as an effective fungicide and nematicide, improving agricultural production, food safety, and security. However, before AgNPs’ mass production and application in agriculture, more research dedicated to the toxic effect of these AgNPs for humans and in vivo study (field trials) should be carried out.

## Figures and Tables

**Table 1:** List of band assignments for FTIR spectra of biogenic AgNPs.

Wave Number (cm^−1^)	Assignment	Reference
3350–3450	Bonded –OH	[20]
2850–2950	CH and CH_2_ stretch	[20]
2800–3000	C-H lipid region	[21]
1620–1670	stretching C=O amide	[22]
1350–1520	Aromatic ether C–O–C, phenolic C–O, and ester C–O–O–C stretching	[23]

**Table 2:** Synthesis of AgNPs from fungi extract and their effect on *A. flavus*.

Fungal Source for AgNPs Mycosinthesis	Concentration of AgNPs	AgNPs Effect on *A. flavus*	References
*P. lilacinum*	54 ppm of nominal metallic Ag	85% of inhibition at 54 ppm	This article
*A. terreus*	n.d.	The microorganisms were inhibited. Concentration is not mentioned.	[27]
*Fusarium oxysporum*	n.d	MIC_50_ = 8 ppm	[24]
*Alternaria* sp.	n.d.	The microorganisms were inhibited. Concentration is not mentioned.	[25]

n.d. = no data; MIC = Minimum inhibitory concentration.

## Data Availability

The data presented in this study are available in the main text, figures, tables.
